# Dysplasie septo optique plus: à propos d’un cas

**DOI:** 10.11604/pamj.2022.42.17.33198

**Published:** 2022-05-09

**Authors:** Lamiaa Chahidi El Ouazzani, Abdelhamid Jadib, Dalal Laoudiyi, Sara Youssef, Kamilia Chbani, Siham Salam, Lahcen Ouzidane

**Affiliations:** 1 Service de la Radiologie Pédiatrique, Hôpital Ibn Rochd, Casablanca, Maroc

**Keywords:** Dysplasie septo-optique, hypoplasie des nerfs optiques, agénésie du septum pellucidum, cas clinique, Septo-optic dysplasia, optic nerve hypoplasia, agenesis of the septum pellucidum, case report

## Abstract

La dysplasie septo optique plus est une pathologie rare qui se voit chez l´enfant. Son diagnostic est radiologique, reposant sur l'imagerie par résonnance magnétique (IRM) cérébrale. Nous rapportons le cas d´un enfant âgé de 2 ans et 4 mois, sans antécédents pathologiques particuliers, qui a consulté pour un retard psycho moteur, un strabisme et des comportements de malvoyance. Un bilan biologique endocrinien explorant la fonction hypothalomo-hypophysaire a été réalisé, ne révélant pas d´anomalie. Le diagnostic de dysplasie septo-optique plus a été retenu sur les données de l´IRM encéphalique, devant l´agénésie du septum pellucidum et du splénium du corps calleux, l´hypoplasie des voies optiques et de la tige pituitaire ainsi que devant l´agénésie de la post hypophyse. Il s´y associait une schizencéphalie fermée. La dysplasie septo-optique est une malformation congénitale rare. Notre objectif est de rappeler sa sémiologie en imagerie et de souligner l´importance de l´IRM pour établir le diagnostic. La dysplasie septo-optique est une entité clinique rare associant classiquement des anomalies encéphaliques de la ligne médiane, une hypoplasie des nerfs optiques et une insuffisance hypophysaire. L´association à des malformations corticales comme la schizencéphalie et la polymicrogyrie désigne le terme dysplasie septo-optique plus. Les progrès de l´imagerie permettent actuellement un diagnostic précoce, ce qui est primordial pour une prise en charge adéquate. L´échographie anténatale peut suspecter la dysplasie, et l´IRM encéphalique confirme le diagnostic.

## Abstract (en)

Septo optic dysplasia plus is a rare disease seen in children. Its diagnosis is radiological, based on brain magnetic resonance imaging (MRI). We report the case of a child aged 2 years and 4 months, with no particular pathological history; who consulted for psychomotor retardation, strabismus and low vision behavior. An endocrine biological assessment exploring the hypothalomo-pituitary function was carried out, revealing no abnormality. The diagnosis of septo-optic dysplasia plus was retained on the brain MRI data, in front of the agenesis of the septum pellucidum and of the splenium of the corpus callosum, the hypoplasia of the optic pathways and of the pituitary stalk as well as in front of the agenesis of the posterior pituitary. It was associated with a closed schizencephaly. Septo-optic dysplasia is a rare congenital malformation. Our objective is to recall its semiology in imaging and to underline the importance of MRI to establish the diagnosis. Septo-optic dysplasia is a rare clinical entity typically involving midline brain abnormalities, optic nerve hypoplasia, and pituitary insufficiency. The association with cortical malformations such as schizencephaly and polymicrogyria denotes the term septo-optic dysplasia plus. Advances in imaging currently allow early diagnosis, which is essential for adequate management. Antenatal ultrasound may suspect dysplasia, and brain MRI confirms the diagnosis.

## Introduction

La dysplasie septo-optique (DSO) ou syndrome de Morsier, est un syndrome malformatif congénital du système nerveux central, caractérisé par un défaut de développement de la ligne médiane, qui comprend une hypoplasie des nerfs optiques, une hypoplasie de l'hypophyse et une agénésie du septum pellucidum, avec ou sans agénésie du corps calleux. Un hypopituitarisme est parfois associé à cette malformation. Des anomalies corticales peuvent s´y associer définissant ainsi une DSO plus. Nous rapportons le cas de notre patient vu l´association à une schizencéphalie fermée il a été diagnostiqué comme dysplasie septo optique plus. L´objectif de notre travail est de montrer la forme de dysplasie septo optique plus qui est une malformation rare caractérisée par la triade sus décrite et de montrer l´importance de l´imagerie et notamment l´IRM dans la confirmation diagnostic.

## Patient et observation

**Information du patient**: un garçon âgé de 2 ans et 4 mois, issu d´une grossesse mal suivie menée à terme, avec accouchement par voie basse, et notion de consanguinité de deuxième degré chez les parents, sans autres antécédents pathologiques particuliers, notamment chez la fratrie. Il présentait un strabisme, des comportements de malvoyance et un retard psychomoteur.

**Résultats cliniques**: il présentait un retard de développement psycho moteur ainsi qu´une baisse de l´acuité visuelle à l´examen ophtalmologique et le fond d´œil a objectivé une atrophie optique bilatérale.

**Figure 1:**
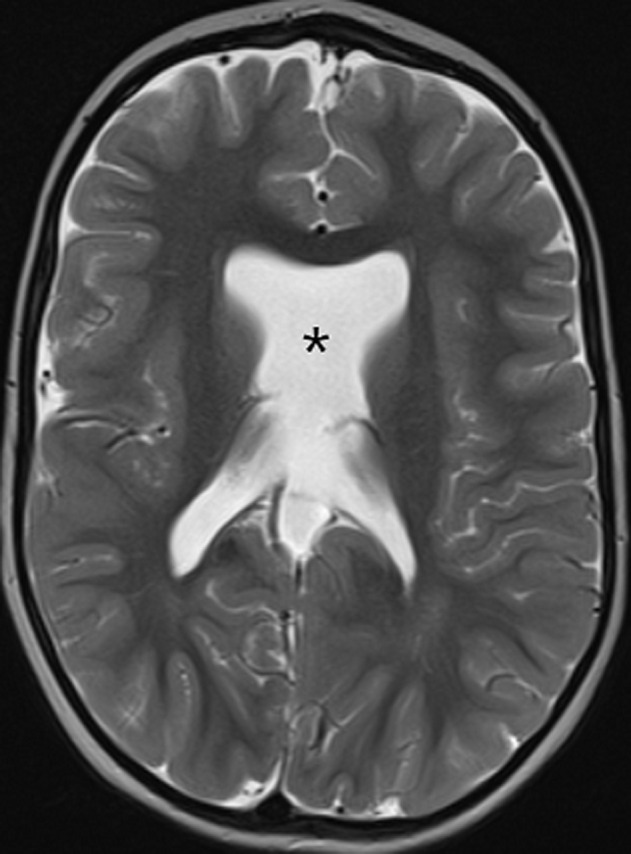
séquence pondérée en T2 en coupe axiale objectivant une absence du septum pellucidum (astérisque)

**Figure 2:**
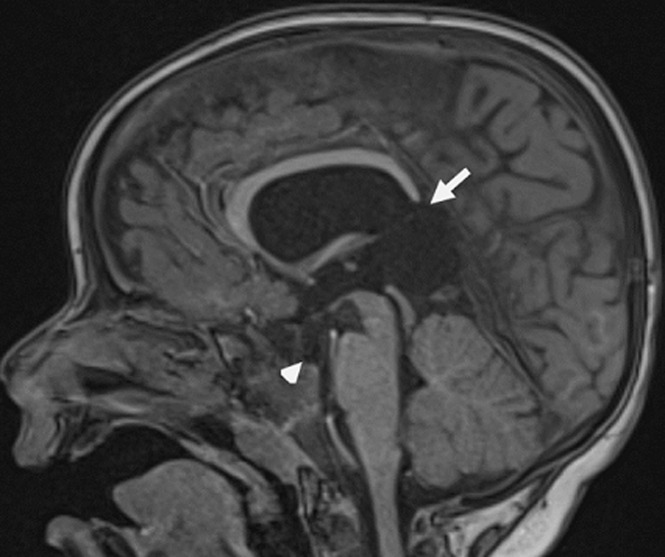
séquence pondérée en T1 en coupe sagittale objectivant une agénésie du splénium du corps calleux (flèche) ainsi qu´une absence de l´hypersignal T1 physiologique de la post hypophyse (tête de flèche)

**Figure 3:**
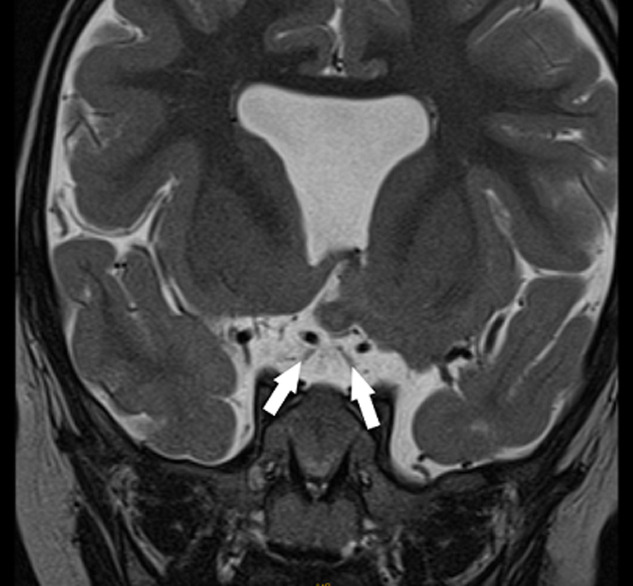
séquence pondérée en T2 en coupe coronale montrant une atrophie des nerfs optiques

**Figure 4:**
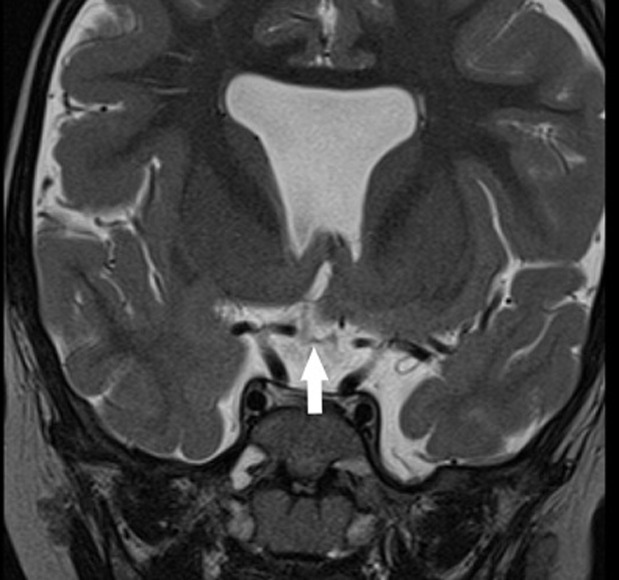
séquence pondérée en T2 en coupe coronale montrant une atrophie du chiasma optique

**Figure 5:**
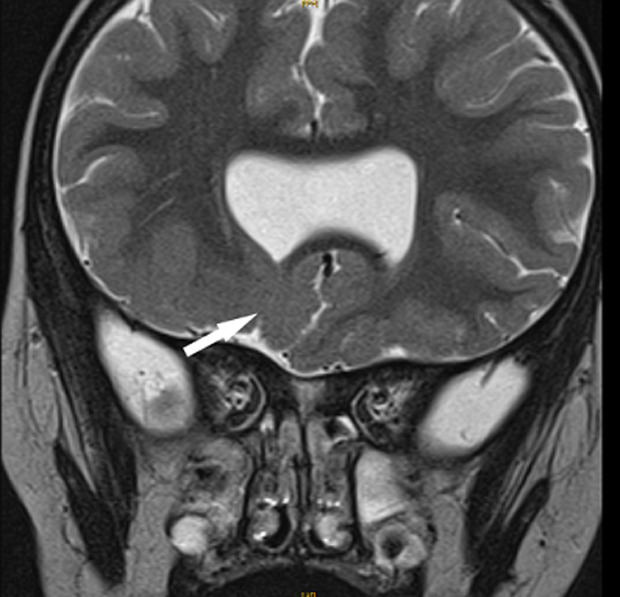
séquence pondérée en T2 en coupe coronale, montrant une schizencéphalie fermée basifrontale droite

**Démarche diagnostique**: une IRM encéphalique, a été réalisée, par une machine 1.5 Tesla. Le protocole comportait des séquences pondérées en T2 en coupe axiale et coronale, une séquence 3D pondérée en T1, une séquence en FLAIR en coupe axiale. Elle a objectivé une agénésie du septum pellucidum (Figure 1), une agénésie du splénium du corps calleux et de la post hypophyse (Figure 2), une hypoplasie des voies optiques (Figure 3 et Figure 4) ainsi qu´un aspect grêle de la tige pituitaire. Il s´y associait une schizencéphalie fermée basi frontale droite (Figure 5). Le bulbe olfactif était d´aspect normal. Le bilan biologique hormonal hypophysaire était sans anomalie. Ces éléments sont compatibles avec une dysplasie septo-optique plus, devant l´association de la triade classique à une schizencéphalie.

**Intervention thérapeutique et suivi**: la prise en charge thérapeutique chez notre patient était essentiellement symptomatique visant à améliorer les troubles d´ordre visuel et intellectuel. Aucun traitement étiologique (éventuelle hormonothérapie) n´a été administré vu la normalité du bilan biologique. Toutefois un bilan hormonal de contrôle évaluant la fonction antéhypophysaire est prévu chez notre malade.

## Discussion

Le terme dysplasie septo optique ou syndrome de « de Morsier » a été décrit pour la première fois par le neurologue français-suisse George De-Morsier, en 1956. Il désigne la triade: hypoplasie des nerfs optiques, dysfonction hypothalamo-hypophysaire, et anomalies de la ligne médiane, incluant l´agénésie du septum pellucidum et du corps calleux [1,2]. Cette pathologie est rare, sporadique dans la plupart des cas avec une prévalence estimée à 1/50000 naissances, sans prédominance de sexe. Peu de facteurs de risque ont été incriminés, comme le diabète sucré maternel, l´infection à cytomégalovirus, l´effet tératogène de certains médicaments comme la quinidine et les habitudes toxiques [3].

Elle est révélée à la naissance en association avec d´autres malformations congénitales. Elle peut se révéler plus tard par un retard staturo-pondéral ou des troubles visuels (parfois indétectable). L´enfant peut présenter un strabisme, un nystagmus ou d´autres anomalies visuelles. Dans la plupart des cas, le diagnostic précoce améliore le pronostic, puisque les anomalies endocriniennes non traitée entrave davantage le développement psychomoteur, et prédisposent le patient aux risques d´hypoglycémie, d´insuffisance surrénalienne et au décès. Le diagnostic doit être suspecté cliniquement chez le nouveau-né devant une hypoglycémie, un ictère, une microcéphalie, une ectopie testiculaire, un nystagmus associé ou non à une anomalie de la ligne médiane, comme la fente labio-palatine. Devant ces circonstances cliniques, un bilan endocrinien et une consultation ophtalmologique sont de mise [4].

L´imagerie joue un rôle important dans le diagnostic. Le scanner permet d´objectiver l´agénésie du septum pellucidum et les anomalies morphologiques associées, intéressant les ventricules latéraux. L´imagerie par résonnance magnétique est l´examen de choix, permettant une meilleure étude des ventricules latéraux et septum pellucidum, des voies optiques et de l´axe hypothalamo-hypophysaire. Elle objective l´agénésie du septum pellucidum, avec un aspect carré des cornes frontales des ventricules latéraux sur les coupes coronales. L´hypoplasie des voies optiques est mieux visible sur les coupes axiales et coronales. Il peut s´y associer une hypoplasie de la tige et/ou de la glande pinéale. Le syndrome de Kallman désigne l´association de la dysplasie septo-optique à une agénésie de bulbes olfactives [5]. Ces éléments décrits en IRM sont suffisants pour retenir le diagnostic de dysplasie septo-optique [4]. L´association à des malformations corticales comme la schizencéphalie et la polymicrogyrie désigne le terme dysplasie septo optique plus [5].

## Conclusion

La dysplasie septo-optique est une entité clinique rare associant classiquement des anomalies encéphaliques de la ligne médiane, une hypoplasie des nerfs optiques et une insuffisance hypophysaire. Les progrès de l´imagerie permettent actuellement d´établir un diagnostic précoce, ce qui est primordial pour une prise en charge adéquate. L´échographie anténatale peut suspecter la dysplasie. L´IRM encéphalique permet de confirmer le diagnostic.
